# P4HA1: an important target for treating fibrosis related diseases and cancer

**DOI:** 10.3389/fphar.2024.1493420

**Published:** 2024-11-06

**Authors:** Xuewen Yang, Dong Zhang, Mengmeng Li, Yingchun Shao, Xiyang Zhang, Yongzhi Xue

**Affiliations:** ^1^ Department of Pharmacology, Institute of Pharmacokinetics and Liver Molecular Pharmacology, Baotou Medical College, Baotou, China; ^2^ Key Laboratory of Basic and Application Research of Beiyao (Heilongjiang University of Chinese Medicine), Ministry of Education, Harbin, China; ^3^ The Affiliated Hospital of Qingdao University, Qingdao Cancer Institute, Qingdao University, Qingdao, China; ^4^ Faculty of Basic Medicine, Chongqing Three Gorges Medical College, Chongqing, China

**Keywords:** prolyl 4-hydroxylase subunit alpha 1, fibrosis, cancer, cardiovascular diseases, mechanism

## Abstract

Fibrosis is significantly associated with a wide variety of diseases and is involved in their progression. Fibrosis activated under the influence of different combinations of factors is considered a double-edged sword. Although there has been much research on organ fibrosis in recent years, a variety of organ fibrosis diseases and cancers are not well controlled in terms of prevention, treatment, and prognosis. Clinical studies still lack exploration and discovery of effective targets for the pathogenesis of organ fibrosis. Prolyl 4-hydroxylase subunit alpha 1 (P4HA1) is a protein kinase and the synthesis and secretion of collagen are related to the sustained activation of P4HA1. As further studies are being conducted, the potential role of P4HA1 in the development of fibrosis-associated diseases and cancer is becoming clear. Consequently, we conducted a systematic review and discussion on the role of P4HA1 in the pathogenesis of various fibrosis-related diseases and cancers. We reviewed the possible strategies of P4HA1 in the diagnosis and treatment of fibrosis-related diseases and cancers, and analyzed its potential relevance as a biomarker in the diagnosis and treatment of fibrosis-related diseases and cancer.

## 1 Introduction

Fibrosis is the result of tissue repair responses following multiple organ injury. Several cell types, including epithelial cells, vascular endothelial cells, and cells of the innate or acquired immune system, participate in fibrosis by secreting factors that recruit and activate fibroblasts to produce extracellular matrix proteins. After tissue damage, local tissue fibroblasts are activated, and the proliferative capacity and extracellular matrix (ECM) synthesis of fibroblasts increase, providing structural support for tissue repair and resulting in repair effects (Henderson et al., 2020; Antar et al., 2023; Yasuma and Gabazza, 2024). Under chronic injury and persistent inflammatory stimuli, the fibrosis process is often uncontrollable, and uncontrolled fibrosis leads to the continued accumulation of ECM components, which may cause tissue structural damage, organ dysfunction, and ultimately organ failure (Henderson et al., 2020; Antar et al., 2023; Yasuma and Gabazza, 2024; Weiskirchen et al., 2019). At present, treatment for organ fibrosis is still in the stage of actively controlling the primary disease (Ngu et al., 2023; Naehrig et al., 2017). Therefore, there is an urgent need to explore the pathogenesis and regulatory network of fibrosis-related diseases, identify effective intervention targets, and develop drugs with targeted precision.

**FIGURE 1:**
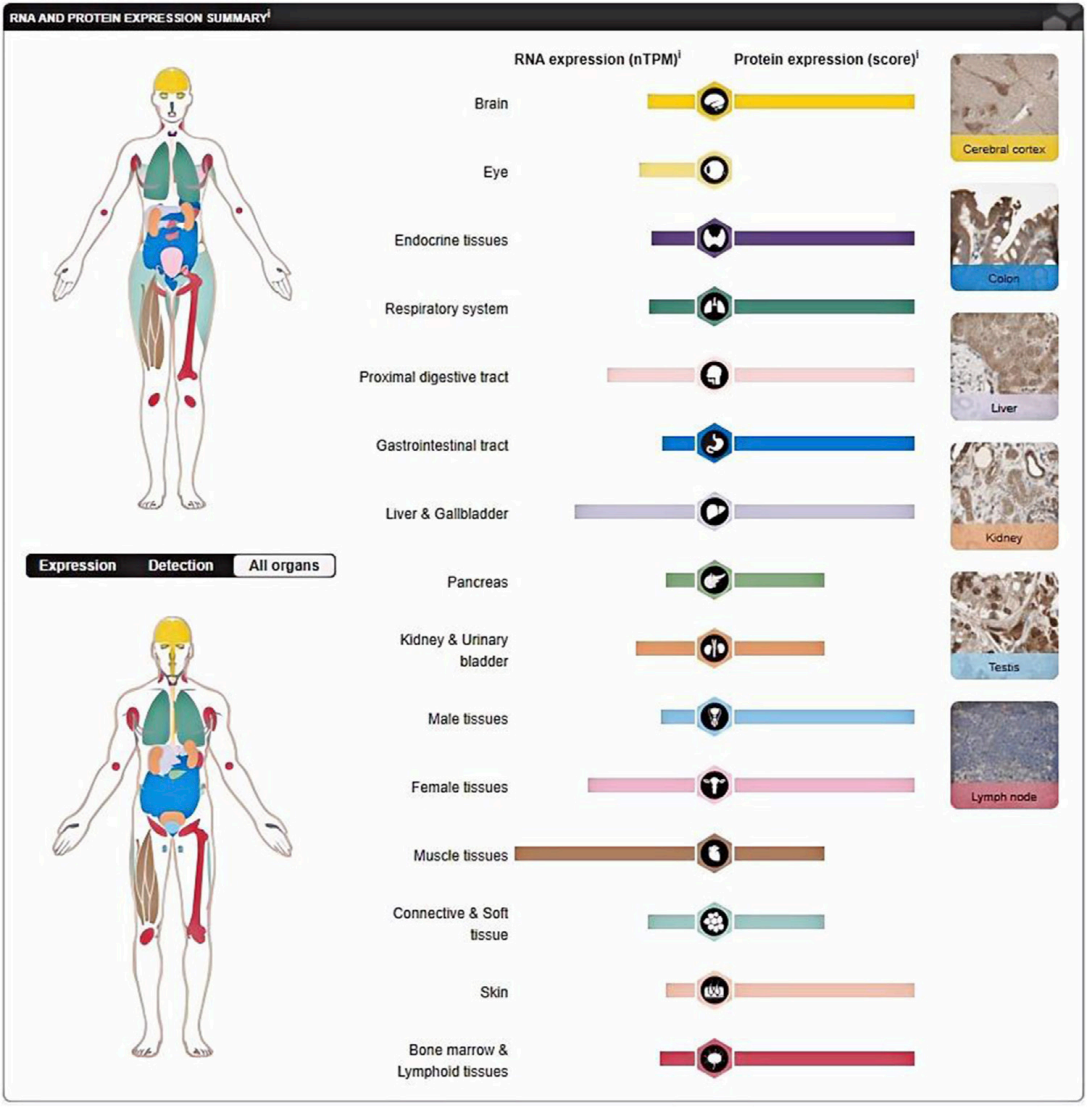
P4HA1 is widely distributed in different tissues. P4HA1 protein is highly expressed in cerebral cortex, nasopharynrynx, and broncius. The expression levels of P4HA1 mRNA are relatively high in muscle tissues, kidney tissues, liver tissues, and female tissues. (https://www.proteinatlas.org/ENSG00000122884-P4HA1/tissue).

Several studies have shown that P4HA1, a key protein involved in collagen synthesis, is a promising therapeutic target for fibrosis-related diseases (Chen et al., 2018; Lou et al., 2017). P4HA1 is composed of two identical alpha subunits and two beta subunits (Zhu et al., 2021; Zou et al., 2017) and plays a central role in the formation and stability of collagen triple helix domains (Kivirikko and Pihlajaniemi, 1998). It plays important roles in various cancers (Table 1), liver diseases, and cardiovascular diseases. P4HA1 is widely distributed in various tissues. For example, P4HA1 mRNA is highly expressed in body parts such as the muscle tissue, kidney, liver, and female tissues, and P4HA1 protein is highly expressed in body parts such as the cerebral cortex, nasopharynx, and bronx (Figure 1). This phenomenon may be attributed to post-transcriptional modifications of RNA (Delaunay et al., 2024), including N6-methyladenosine (m6A) and N5-methylcytosine (m5C), as well as post-translational modifications of proteins (Lee et al., 2023), such as phosphorylation and ubiquitination. Splicing, capping, and tailing processes after transcription of RNA may affect the stability of mRNA, potentially leading to elevated transcription levels of mRNA and diminished protein expression (Hao et al., 2024; Gilbert and Nachtergaele, 2023). Post-translational modifications of proteins affect a number of key biological processes, including expression, localization, and enzyme activity (Wang et al., 2023). Consequently, an increase in protein stability and a reduction in the degradation rate may result in a reduction in mRNA transcription levels, while protein expression levels remain elevated. A deeper study on the role of P4HA1 in fibrosis will broaden the perspective of potential targets for treatment. In this article, we discuss the regulatory factors of P4HA1 expression and the signaling pathways involved in diseases caused by P4HA1.

**TABLE 1:** Studies reporting P4HA1 in cancer.

Disease model	P4HA1 expression	Main function	References
Colorectal Cancer	↑	P4HA1 knockdown inhibits colon cancer cell proliferation and reduces stemness	Xu et al. (2019); Li et al. (2020); Chen et al. (2021)
Gliomas	↑	P4HA1 promotes GBM cell migration and invasion	Shin et al. (2023); Tanaka et al. (2020): Gawel et al. (2019)
Lung Cancer	↑	P4HA1 promotes lung adenocarcinoma cell invasion and metastasis	Yang et al. (2024)
Prostate Cancer	↑	P4HA1 promotes prostate cancer cell growth, tumor progression, and cancer cell stemness	Zhou et al. (2020); Yang et al. (2023)
Pancreatic Cancer	↑	P4HA1 promotes PDAC cell proliferation, drug resistance, and stemness	Zhang et al. (2023); Chakravarthi et al. (2014); Walenta et al. (2004)
Breast Cancer	↑	P4HA1 promotes breast cancer cell metastasis, invasiveness and stemness	Hu et al. (2020); Cao et al. (2019); Li et al. (2023)
Esophageal Cancer	↑	P4HA1 promotes esophageal cancer progression	Hollern et al. (2014)
Hepatocellular Carcinoma	↑	P4HA1 promotes the proliferation of liver cancer cells	Polley et al. (2021)
Ovarian Cancer	↑	P4HA1 promotes ovarian cancer cell migration and invasion	Li et al. (2022); Gou et al. (2023a)

↑: GDF11 expression increased.

## 2 P4HA1 and cancer

Cancer is driven by genetic changes that disrupt the survival, proliferation, and spread of cancer cells (Kiri and Ryba, 2024). In 2020, there were a total of 4,546,400 new cases of cancer and 2,992,600 deaths in China, accounting for 25.1% and 30.2% of global cases, respectively (He et al., 2024). The noncancerous components of tumor tissues (including fibroblasts, inflammatory cells, and ECM) play a crucial role in tumorigenesis and cancer progression. This provides a mutagenic environment that allows cancer cells to develop, facilitating their survival, expansion, and invasiveness (Landolt et al., 2022; Mallikarjuna et al., 2022; Nicolini et al., 2023). This presents serious difficulties in the treatment of cancers, such as the emergence of immunotherapy and medication resistance (Naik and Leask, 2023; Xiao and Yu, 2021). Collagen promotes the infiltration, invasion, migration, and angiogenesis of malignant tumors by reshaping the ECM and influencing the tumor microenvironment (Xu et al., 2019; Su and Karin, 2023; Necula et al., 2022). P4HA1 is responsible for producing 4-hydroxyproline at the Yaa position of the Gly Xaa Yaa repeat sequence in collagen, which is necessary for the formation of the collagen triple helix structure (Taga et al., 2014). Previous studies have shown that increased P4HA1 expression is associated with poor prognosis in some solid cancers, such as pancreatic cancer, colon cancer, high-grade glioma, breast cancer, prostate cancer, and lung cancer (Zhou et al., 2023; Zhao and Liu, 2021; Li et al., 2020; Chen et al., 2021).

### 2.1 Colon cancer

Colorectal cancer (CRC) is the third most common malignant tumor of new cancer cases worldwide (Ionescu et al., 2023; Aljama et al., 2023). The metastasis of CRC is significantly correlated with matrix deposition and remodeling (Shin et al., 2023), indicating that P4HA1 may also have carcinogenic effects in CRC. Tanaka et al. (2020) found through tissue analysis of clinical cases of 599 patients with stage I or II CRC that P4HA1 is mainly expressed in the malignant epithelial components of CRC. In addition, Gawel et al. (2019) found that the combination of P4HA1 with tripartite motif-containing 28 (TRIM28), procollagen-lysine, 2-oxoglutarate 5-dioxygenase 1 (PLOD1) and carcinoembryonic antigen-related cell adhesion molecule 5 (CEACAM5) proteins in the plasma of 80 newly diagnosed CRC patients and 80 healthy controls can serve as potential biomarkers for early diagnosis of colorectal cancer. This indicates that P4HA1 plays an important role in the occurrence, development, and diagnosis of CRC. However, the mechanism of action of P4HA1 in CRC is still unclear.

**FIGURE 2:**
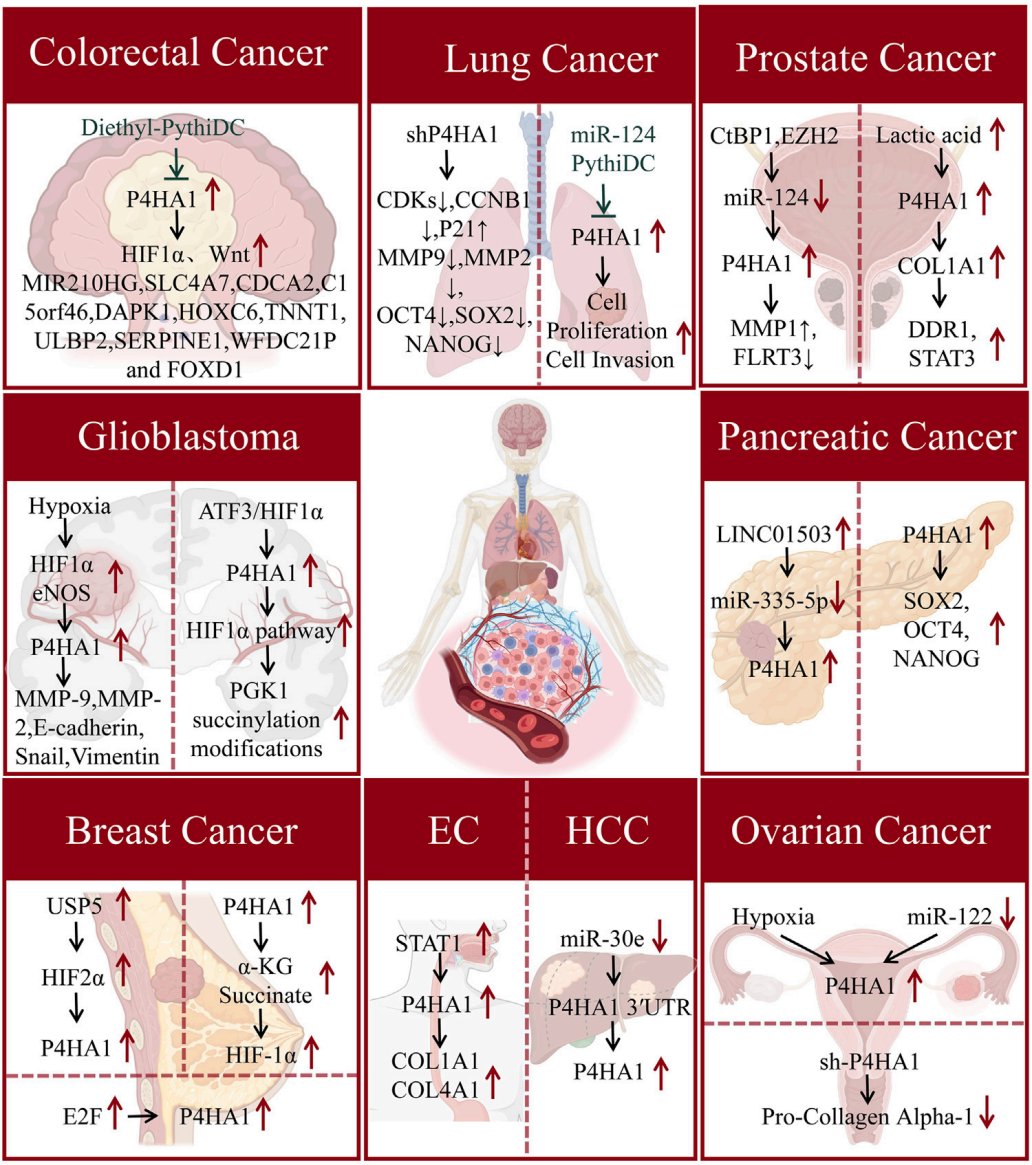
The mechanism of action of P4HA1 in various cancers. P4HA1 is involved in regulating the proliferation, migration, and invasion of various cancer cells. The figure summarizes the mechanism by which P4HA1 contributes to the occurrence and progression of cancers in the manuscript.

Zhang et al. (2021) found that P4HA1 expression can stabilize hypoxia inducible factor-1 alpha (HIF1α) and activate the Wnt signaling pathway, promoting the proliferation of CRC cells. Chen et al. found through gene expression profiling analysis using the Cancer Genome Atlas (TCGA) that the risk signal of P4HA1 related genes in CRC consists of 11 genes, including MIR210HG, solute carrier family 4 member 7 (SLC4A7), cell division cycle associated 2 (CDCA2), death associated protein kinase 1 (DAPK1), homeobox C6 (HOXC6), Troponin T 1 (TNNT1), UL16 binding protein 2 (ULBP2), serine protease inhibitor clade E member 1 (SERPINE1), WFDC21P, and forkhead box D1 (FOXD1) (Chen et al., 2021). In addition, Agarwal et al. found that P4HA1 is highly expressed in CRC tissues and promotes tumor cell proliferation, invasion, migration, and tumor growth. And diethyl pyhidc can inhibit the progression of invasive CRC by acting on P4HA1 (Agarwal et al., 2020) (Figure 2). The above research progress suggests that P4HA1 may serve as an early diagnostic biomarker and therapeutic target for CRC, but its pathogenic mechanism in CRC is still unknown.

### 2.2 Gliomas

Glioma is the most common malignant tumor of the central nervous system in adults and is divided into different subtypes. Among them, glioblastoma multiforme (GBM) has the highest number and the strongest lethality (Uddin et al., 2022). The ECM is significantly correlated with the stemness and invasion of glioma cells. Cescon et al. (2023) found that collagen VI is involved in maintaining the stem cell-like properties of GBM cells and promoting invasive transcriptional programs for cancer cell proliferation and survival. P4HA1 is a key rate limiting protein in the process of collagen synthesis. Hu et al. found that P4HA1 expression is upregulated in gliomas. The high expression of P4HA1 is associated with the malignancy of glioma and can serve as a prognostic indicator for high-grade glioma patients (Hu et al., 2017). The hypoxic microenvironment affects the invasiveness of cancer cells., Hypoxia promotes cancer cell migration and invasion through the L-Arg/P4HA1 axis in GBM (Zhu et al., 2021). In addition, Yang et al. (2024) found that in GBM cells, P4HA1 enhances PK1 succinylation by affecting succinate concentration, and succinylation inhibits proteasomal degradation of phosphoglycerate kinase 1 (PGK1), significantly increasing aerobic glycolysis to produce lactate. Overexpression of activating transcription factor 3 (ATF3) inhibits the binding of HIF1α to the P4HA1 promoter region under hypoxic conditions, suppressing immune response and tumor growth (Figure 2). Chitosan gelatin microspheres loaded with P4HA1 siRNA can significantly inhibit the proliferation, metastasis, glial layer formation, and protein levels of stromal markers (N-cadherin, vimentin) and epithelial mesenchymal transition (EMT) transcription factors (Snail, Slug, Twist1) in glioma cells (Zhou et al., 2024). The above research suggests that P4HA1 correlates significantly with the expression of transcription factor HIF1α in GBM. Developing therapeutics targeting P4HA1 and HIF1α may be the way to go for treating GBM in the future.

### 2.3 Lung cancer

Lung cancer is the leading cause of cancer-related death worldwide. Histologically, lung cancer can be divided into small-cell lung cancer (SCLC) and non-small cell lung cancer (NSCLC) (Jha et al., 2024). At present, the main treatment strategies for lung cancer include molecular targeted therapy, photothermal therapy, and immunotherapy (Feng and Zhang, 2023; Alduais et al., 2023; Lahiri et al., 2023). Research has shown that P4HA1 is essential for the growth and invasion of lung cancer cells, indicating that P4HA1 may be an effective therapeutic target for lung adenocarcinoma (Zhao and Liu, 2021). Zhou et al. (2020) found that the expression of P4HA1 was upregulated by 40% in tumor tissues compared to normal tissues of lung adenocarcinoma. In addition, both P4HA1 mRNA and protein are upregulated in NSCLC. Further research has found that P4HA1 promotes the invasion and metastasis of lung adenocarcinoma tumor cells by affecting EMT and matrix metalloproteinases (MMPs) expression (Ning et al., 2021). MicroRNAs (miRNAs) are a class of non-coding RNAs with a length of approximately 21 nucleotides, and studies have shown that the expression of some miRNAs is dysregulated in NSCLC (Yang et al., 2023; Lobera et al., 2023; Rajakumar et al., 2023). Robinson et al. found that overexpression of miR-124 can significantly inhibit the expression of P4HA1 protein in lung cancer cells, resulting in tumor-suppressive effects (Robinson et al., 2021).

The above studies indicate that P4HA1 plays an important role in the disease progression of lung cancer (Figure 2). Li et al. (2020) found through survival analysis that lung cancer patients with high P4HA1 have a poorer clinical prognosis. Targeting P4HA1 is a promising strategy for treating lung cancer. Therefore, there is an urgent need to develop small molecule inhibitors targeting lung cancer cell P4HA1. Robinson et al. discovered that the small molecule inhibitor PythiDC of P4HA1 can significantly inhibit the cell viability and invasion ability of lung cancer cells (Robinson et al., 2021). The above research indicates that P4HA1 plays a key role in the pathogenesis and prognosis of lung cancer. Furthermore, P4HA1 inhibitors have the potential to become a treatment for lung cancer. However, P4HA1 has not been reported in lung fibrosis, such as idiopathic pulmonary fibrosis. This suggests that research on P4HA1 in pulmonary fibrosis-related diseases is still lacking and that in-depth studies are highly valuable.

### 2.4 Prostate cancer

Prostate cancer (PCa) is a widespread cancer, which mainly affects men, with a high incidence rate and mortality. It is the second most common cancer in men, after lung cancer (Zhang et al., 2023). In general, there are no typical symptoms in the early stages of PCa, and most newly diagnosed PCa patients are often in the advanced stage. In addition, prostate biopsy is considered the gold standard for the diagnosis of PCa. Currently, there is a lack of relevant biomarkers for the diagnosis and prognosis of PCa. ECM is a major component of the tumor environment, promoting the establishment of pre-invasive behavior. A number of studies have shown that P4HA1 expression is associated with the progression of PCa. Chakravarthi et al. (2014) found that P4HA1 expression was significantly increased in metastatic prostate cancer tissues. Further mechanistic studies have shown that miR-124 regulates prostate cancer cell growth and tumor progression by acting on the expression of P4HA1 and MMP1. Lactic acid is one of the most abundant environmental metabolites in tumors, and its levels are significantly correlated with cancer metastasis in cancer patients (Walenta et al., 2004). Ippolito et al. (2024) found that lactate secreted by cancer-associated fibroblasts promotes an increase in alpha-ketoglutarate (α-KG) in prostate cancer cells, activating α-KG dependent P4HA1 to increase collagen hydroxylation, thereby inducing stemness and invasive features of prostate cancer cells. The above research progress indicates a significant correlation between P4HA1 and cancer metastasis in PCa.

### 2.5 Pancreatic cancer

Pancreatic cancer is the leading cause of cancer-related death worldwide. At present, clinical treatment for pancreatic cancer is mainly divided into surgery and chemotherapy (Kolbeinsson et al., 2023; Wood et al., 2022; Milella et al., 2022). However, there is still a lack of specific therapeutic targets and biomarkers for pancreatic cancer. Hu et al. (2020) analyzed tumor and normal samples in different datasets and showed that P4HA1 was significantly overexpressed in multiple pancreatic cancer datasets. Ductal adenocarcinoma of the pancreas (PDAC) is the main type of pancreatic cancer. After overexpression of P4HA1, KEGG pathway enrichment analysis showed a significant correlation with the HIF-1 signaling pathway. Research has found that P4HA1 enhances the stability of HIF1α, promotes glycolytic activity in PDAC cells, induces cancer cell proliferation, drug resistance, and stemness (Cao et al., 2019). Cao X. et al. (2023) found that ectopic expression of P4HA1 increased the levels of cancer stem cell-associated proteins [sex-determining region (SOX2), octamer-binding transcription factor 4 (OCT4), and nanog homeobox (NANOG)] in pancreatic ductal adenocarcinoma cells. However, the specific mechanism and key proteins of P4HA1 in the occurrence and malignant progression of pancreatic cancer are still unclear, which deserve further discussion. Hu et al. found that LINC01503/miR-335-5p is the most promising upstream regulation axis that affects P4HA1 in pancreatic cancer through correlation analysis (Hu et al., 2020). Previous studies have demonstrated that P4HA1 plays a significant role in the pathogenesis of pancreatic cancer. However, further investigation is required to elucidate the disease mechanisms and to develop targeted therapeutic agents.

### 2.6 Breast cancer

Breast cancer (BC) is a common malignant tumor in women globally. Collagen deposition is significantly related to the progress and metastasis of BC (Herrera-Quintana et al., 2024; Papanicolaou et al., 2022; Li et al., 2023). However, at present, the specific mechanism of BC is still unclear. Further clarification of new and more specific biomarkers for the diagnosis, prognosis, and risk prediction of BC is of great significance to achieve personalized treatment, improve treatment, and prevent overtreatment, undertreatment, and incorrect treatment. The regulation of P4HA1 has a significant impact on the prognosis of BC patients (Li et al., 2020; Murugesan and Premkumar, 2021). Hollern et al. (2014) found that E2F transcription factors promote the metastasis of breast cancer, while E2F downstream target genes include Vegfa, Bmp4, Cyr61, and P4HA1, suggesting that P4HA1 may regulate collagen deposition and participates in the regulation of cancer metastasis and invasion. In addition, ubiquitin-specific peptidase 5 (USP5) is highly expressed in breast cancer. USP5 deubiquitination modifies HIF2α, and protects HIF2α from ubiquitin-proteasome degradation, thus promoting the transcription of HIF2α target genes, such as P4HA1, solute carrier family 2 member 1 (SLC2A1), PLOD2 and vascular endothelial growth factor A (VEGFA), providing a potential therapeutic target for BC (Huang et al., 2022) (Figure 2). Triple-negative breast cancer (TNBC) is the most aggressive and heterogeneous of all BC subtypes (Polley et al., 2021; Vagia et al., 2020; Rigiracciolo et al., 2020). The activation of the HIF-1 pathway in TNBC is at least partially regulated by P4HA1, promoting the stemness of cancer cells. In addition, elevated expression of P4HA1 is associated with poor prognosis and chemotherapy resistance in TNBC patients. The combination of P4Hi and chemotherapy drug doxorubicin can overcome TNBC chemotherapy resistance (Xiong et al., 2018).

### 2.7 Other cancers

Previous studies have elucidated the function and operational process of P4HA1 in colon cancer, gliomas, lung cancer, prostate cancer, and pancreatic cancer. What is the function of P4HA1 in other types of cancers? The ECM is the main component of the tumor microenvironment. Collagen can promote the invasion and migration of malignant tumors, and P4HA1 is a key enzyme of collagen. Li et al. (2022) inferred that P4HA1 may play an important role in the tumorigenesis of clear cell renal cell carcinoma (RCC) and may be a prognostic biomarker and therapeutic target for various malignancies, including RCC. Gou et al. (2023a) found that the expression of P4HA1 is related to the differentiation degree, location, lymph node metastasis, and tumor lymph node metastasis staging of esophageal squamous cell carcinoma. And it was discovered that P4HA1 is activated by STAT1 transcription, thereby promoting the progression of esophageal cancer (EC) (Gou et al., 2023b). Hepatocellular carcinoma (HCC) is the leading cause of cancer-related deaths around the world, particularly in populations in Asia and Africa. The expression level of miR-30e is reduced in liver cancer tissues. Further research has found that miR-30e can reduce the expression of P4HA1 at both mRNA and protein levels, inhibiting the proliferation of liver cancer cells (Feng et al., 2016).

Ovarian cancer is an invasive disease, and the deposition of collagen is significantly correlated with the invasion, prognosis, and metastasis of ovarian cancer (Akinjiyan et al., 2024; Lyu and Feng, 2021; Ho et al., 2021). Platinum-based chemotherapy is the cornerstone of ovarian cancer treatment, but the resistance of ovarian cancer cells to platinum-based chemotherapy seriously affects the prognosis and survival of ovarian cancer patients. Song et al. observed that hypoxia can significantly upregulate the mRNA and protein expression of P4HA1/2, while knocking down P4HA1/2 can significantly inhibit collagen secretion, migration, and metastasis of ovarian cancer cells (Song et al., 2023). In addition, miR-122 has tumor-suppressive effects on various cancers. Duan et al. (2018) found that miR-122 inhibited the migration, invasion, and EMT of ovarian cancer cells by downregulating P4HA1. MiR-122 and P4HA1 may be potential diagnostic markers and therapeutic targets in ovarian cancer.

Levofloxacin has broad-spectrum anticancer activity, and its combination with cisplatin further enhances the cytotoxicity of cancer cells by promoting apoptosis (He et al., 2022a). Levofloxacin prevents DNA replication in bacteria by inhibiting the activity of DNA helicase. He et al. (2022b) found that levofloxacin significantly inhibited cancer cell proliferation, colony formation, and xenograft tumor growth by blocking the G2/M cell cycle and promoting cell apoptosis. Additionally, P4HA1 is enriched in differentially downregulated genes. P4HA1 mediated high collagen deposition plays a crucial role in the tumor microenvironment and progression, and new therapeutic strategies or small-molecule inhibitors targeting collagen synthesis are being developed, which will be an important direction for future cancer research.

## 3 P4HA1 and cardiovascular diseases

Cardiovascular disease is the leading cause of morbidity and mortality worldwide. Fibrosis is a common feature of cardiovascular diseases. Cardiovascular fibrosis represents the activation of repair mechanisms for damaged organs. However, prolonged and uncontrolled activation of these repair mechanisms can result in excessive remodeling and hardening of the ECM, leading to impaired cardiac function and ultimately heart failure (Poe A et al., 2023; Ravassa Set al., 2023). The following section will further discuss the role and specific mechanisms of P4HA1 in the context of cardiovascular disease fibrosis.

### 3.1 Atherosclerosis

Atherosclerosis is the main cause of cardiovascular disease, which is characterized by the accumulation of lipids and fiber elements in the great arteries. Collagen synthesis by vascular smooth muscle cells (VSMCs) is very important in atherosclerosis because it affects plaque stability (Grootaert and Bennett, 2021; Miano et al., 2021; Zhai et al., 2022). miRNAs play an important role in cardiovascular diseases (Han et al., 2021; Bian et al., 2021; Gao et al., 2022). Chen et al. (2018) found a negative correlation between collagen and VSMC content in plaques and miR-124-3p levels. MiR-124-3p inhibits VSMC collagen synthesis by directly targeting P4HA1, which may reduce the stability of atherosclerotic plaques. Low shear stress and oscillatory shear stress can affect the size and phenotype of coronary atherosclerotic lesions. P4HA1 overexpression increases the fiber cap thickness and collagen content of carotid plaques induced by low shear stress and oscillatory shear stress, leading to a significant increase in the size of atherosclerotic plaques (Cao et al., 2016). Plaque rupture is the most common cause of coronary artery occlusion, which can lead to acute coronary syndrome. IL-6 significantly increased the phosphorylation of RAF, mitogen-activated protein kinase (MEK)1/2 and extracellular signal-regulated kinase (ERK) 1/2, and the transcription factor c-Jun mediated the reduction of P4HA1 transcription, downregulated the expression of P4HA1, thereby destroying the stability of mouse atherosclerotic plaques (Zhang et al., 2012). Melatonin is an endogenous neurohormone primarily secreted by the pineal gland, with multiple physiological functions. Li et al. (2019) found that melatonin increased Akt phosphorylation and transcription activation of specific protein 1 (Sp1), which binds to P4HA1 promoter, induces P4HA1 expression, and enhances the stability of atherosclerotic plaques in ApoE −/− mice.

### 3.2 Myocardial infarction

Myocardial infarction (MI) is the main cause of global incidence rate and mortality, and also the main cause of heart failure (HF) (Groenewegen et al., 2020; Frantz et al., 2022). The significant loss of myocardial cells and excessive deposition and arrangement of ECM after myocardial infarction leads to serious consequences such as cardiac fibrosis (Yin et al., 2023). Fischer et al. (2024) found that cellular communication network factor (CCN)1 plays a crucial role in scar formation after myocardial infarction, guiding the appropriate arrangement of extracellular matrix collagen components in mature scars - shaping the mechanical properties that support their structural stability. Further research has found that the absence of CCN1 reduces the expression of collagen processing and stabilizing enzymes (i.e., P4HA1, Procollagen-lysine 2-oxyglutarate 5-dioxygenase (PLOD)1, and PLOD2). CCN1 gene knockout mice showed higher ECM structural complexity in the scar area after myocardial infarction, including reduced local arrangement and increased curvature of collagen fibers, as well as a 90% decrease in tissue consistency, packaging, and size of collagen fibrils. The above studies indicate that P4HA1 plays an important role in the synthesis and arrangement of collagen during the fibrosis process after myocardial infarction.

### 3.3 Diabetic cardiomyopathy

Diabetic cardiomyopathy (DCM) is a serious complication of diabetes (Shao et al., 2022), leading to cardiac fibrosis, even heart failure and other serious consequences (Nakamura et al., 2022). Zhao et al. found that liraglutide can upregulate the expression levels of CD36 and p-JNK, enhance the DNA-binding activity of activator protein (AP)-1 to P4HA1, thereby downregulating P4HA1 expression and reducing myocardial fibrosis (Zhao et al., 2019). This provides a new therapeutic target for heart fibrosis caused by diabetic cardiomyopathy.

**FIGURE 3:**
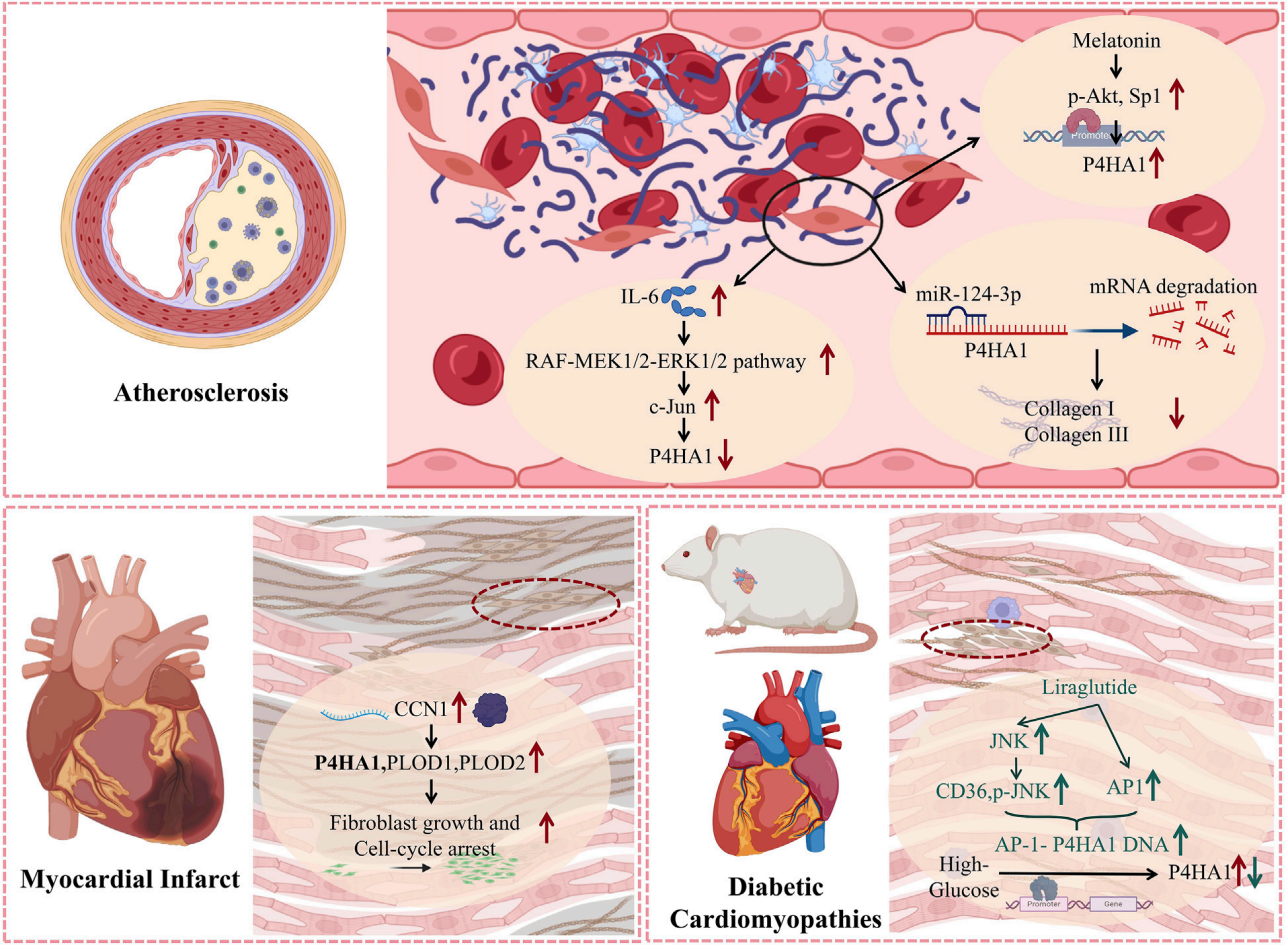
The mechanism of action of P4HA1 in various cardiovascular diseases. P4HA1 is involved in regulating the proliferation of fibroblasts and the synthesis of collagen in various cardiovascular diseases. The molecular processes of P4HA1 involvement in cardiovascular disease discussed in the text are summarized in this figure.

The above results indicate that P4HA1 mediates the synthesis and secretion of collagen, which influences the stability of atherosclerotic arterial plaques and the process of cardiac fibrosis in myocardial infarction and diabetic cardiomyopathy (Figure 3).

## 4 P4HA1 and other diseases

Non-alcoholic fatty liver disease (NAFLD) is currently the most common liver disease and a global disease that threatens human health. The progression of NAFLD may ultimately result in fibrosis and cirrhosis (Pouwels et al., 2022). In multiple studies, it has been found that P4HA1 is a hub gene in NAFLD, and its expression is downregulated by 95% in NAFLD (Jiang H. et al., 2023a). Cao J. et al. (2023) found a significant correlation between P4HA1 and neutrophils. The above research suggests that P4HA1 may participate in the disease progression of NAFLD by participating in cellular metabolism and inflammatory responses. In addition, P4HA1 is also involved in the process of liver fibrosis. Li et al. found that overexpression of miR-122 in hepatic stellate cells significantly reduced the expression of P4HA1 by targeting the binding site of P4HA1 mRNA 30-UTR, leading to decreased collagen maturation and ECM generation, and inhibited liver fibrosis (Li et al., 2013; Lou et al., 2017).

Periodontal disease is a multifactorial chronic disease. It is usually accompanied by a hypoxic environment, which affects metabolic activation and exacerbates pathological and physiological conditions (Gou et al., 2022). The extracellular matrix of periodontal connective tissue comprises a substantial proportion of type I collagen. Morimoto et al. found that hypoxia culture stimulates upregulation of P4HA1 expression in periodontal ligament cells, increasing collagen levels (Morimoto et al., 2021).

The airway remodeling in asthma airway inflammation is caused by the deposition of collagen on the airway wall. Chelidonium majus may alleviate airway remodeling induced by ovalbumin in asthmatic rats by affecting the expression of P4HA1 (Wang et al., 2024). The above research results indicate that P4HA1 could be used as one of the targets for developing therapeutic drugs for airway inflammation.

**FIGURE 4:**
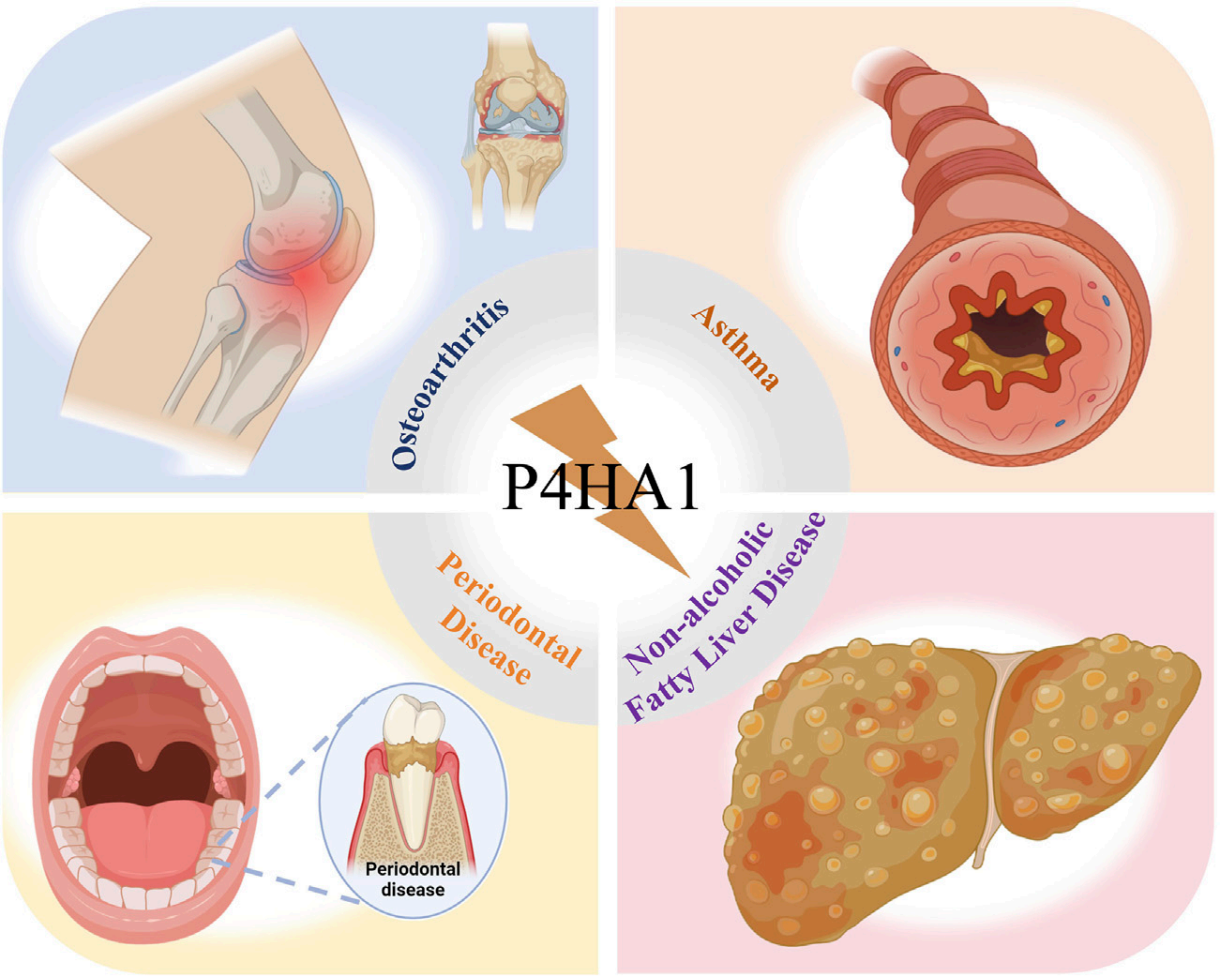
P4HA1 is significantly correlated with osteoporosis, asthma, periodontal disease, and NAFLD.

Osteoarthritis (OA) is the most common type of arthritis. In OA, the composition and viscoelasticity of the ECM produced by chondrocytes undergo alterations (Hodgkinson et al., 2022). According to reports, P4HA1 disrupts the structure of the vascular basement membrane by inhibiting collagen synthesis (Zhou et al., 2017). Jiang P. et al. (2023) found that miRNA-1 treatment led to a decrease in the expression levels of P4HA1 and aggrecan (ACAN), delaying articular cartilage degeneration (Figure 4).

## 5 Summary

According to existing research, the role of P4HA1 in pancreatic cancer, colon cancer, high-grade glioma, breast cancer, prostate cancer, lung cancer and other cancers has been preliminarily verified. However, the role of P4HA1 in cardiovascular diseases such as myocardial infarction, ischemia-reperfusion, and heart failure with preserved ejection fraction remains to be explored. Therefore, it is necessary to further expand the research scope and explore the specific roles and mechanisms of P4HA1 in different types of cardiovascular diseases. P4HA1 is expressed in various organs. This explains its relationship with cancer and cardiovascular disease. Overall, research on the role of screening small-molecule drugs targeting P4HA1 in organ fibrosis diseases and cancer is limited. Therefore, based on current research results, more evidence is needed to apply strategies for treating organ fibrosis by inhibiting the expression of P4HA1 gene and protein. In addition, due to the limitations of research on the mechanism of P4HA1 fibrosis in cardiovascular diseases. Therefore, future research should explore the mechanism of action of P4HA1 through various methods such as cell experiments, animal models, clinical cases, and comprehensively analyze other related genes and signals to understand the role of P4HA1 in fibrosis in cardiovascular diseases. We hope that with the continuous advancement of technology and the continuous development of research, the potential of P4HA1 in treating cardiovascular and cerebrovascular diseases will gradually be discovered and realized.
